# Subfascial‐located contraceptive devices requiring surgical removal

**DOI:** 10.1186/s40834-021-00158-5

**Published:** 2021-05-03

**Authors:** Justin E. Hellwinkel, Matthew W. Konigsberg, Johana Oviedo, Paula M. Castaño, R. Kumar Kadiyala

**Affiliations:** 1 Department of Orthopedics, Columbia University Irving Medical Center, 622 W 168th St PH 11 – Center, NY 10032 New York, USA; 2 Department of Obstetrics and Gynecology, New York University Langone Health, 550 First Avenue, NY 10016 New York, USA; 3 Department of Obstetrics and Gynecology, Columbia University Irving Medical Center, 622 W 168th St, NY 10032 New York, USA

**Keywords:** Contraceptive implant removal, Etonogestrel complication, Subdermal etonogestrel, Subfascial implant removal

## Abstract

**Background:**

Subdermal etonogestrel implants are highly effective contraceptive methods. Despite standardization of insertion technique by the manufacturer, some implants are inadvertently placed too deeply within or below the plane of the biceps brachii fascia. Placement of these implants in a deep tissue plane results in more difficult removal, which is not always possible in the office setting. In rare cases, surgical removal by an upper extremity surgeon is warranted.

**Case presentation:**

Here we present 6 cases of etonogestrel implants located in a subfascial plane requiring removal by an upper extremity surgeon. Implants were all localized with plain radiography and ultrasound prior to surgical removal. All cases had implants located in the subfascial plane and one was identified intramuscularly. The average age was 28 years (19–33) and BMI was 24.0 kg/m^2 (19.1–36.5), with the most common reason for removal being irregular bleeding. The majority of cases (5/6) were performed under monitored anesthesia care with local anesthetic and one case utilized regional anesthesia. All implants were surgically removed without complication.

**Conclusions:**

Insertion of etonogestrel contraceptive implants deep to the biceps brachii fascia is a rare, but dangerous complication. Removal of these implants is not always successful in the office setting and referral to an upper extremity surgeon is necessary to avoid damage to delicate neurovascular structures for safe removal.

## Background

Subdermal etonogestrel implants are highly effective contraceptive methods [1]. The manufacturer recommends subdermal placement of the implant overlying the triceps muscle in the medial upper arm using a preloaded insertion implant [2]. All healthcare practitioners providing insertions are trained in proper placement techniques, including immediate palpation to confirm subdermal location, and in-office removal techniques. Deep placement can make removal particularly challenging.

Our institution is a referral center for complex implant removals. After review of plain radiographs (XR) to confirm upper-arm location of non-palpable implants, evaluation and in-office high-frequency ultrasonography (US), we refer patients with deeply-located implants not amenable to in-office removal to a single upper extremity orthopaedic surgeon for operative removal.

## Case Series

**Fig. 1:**
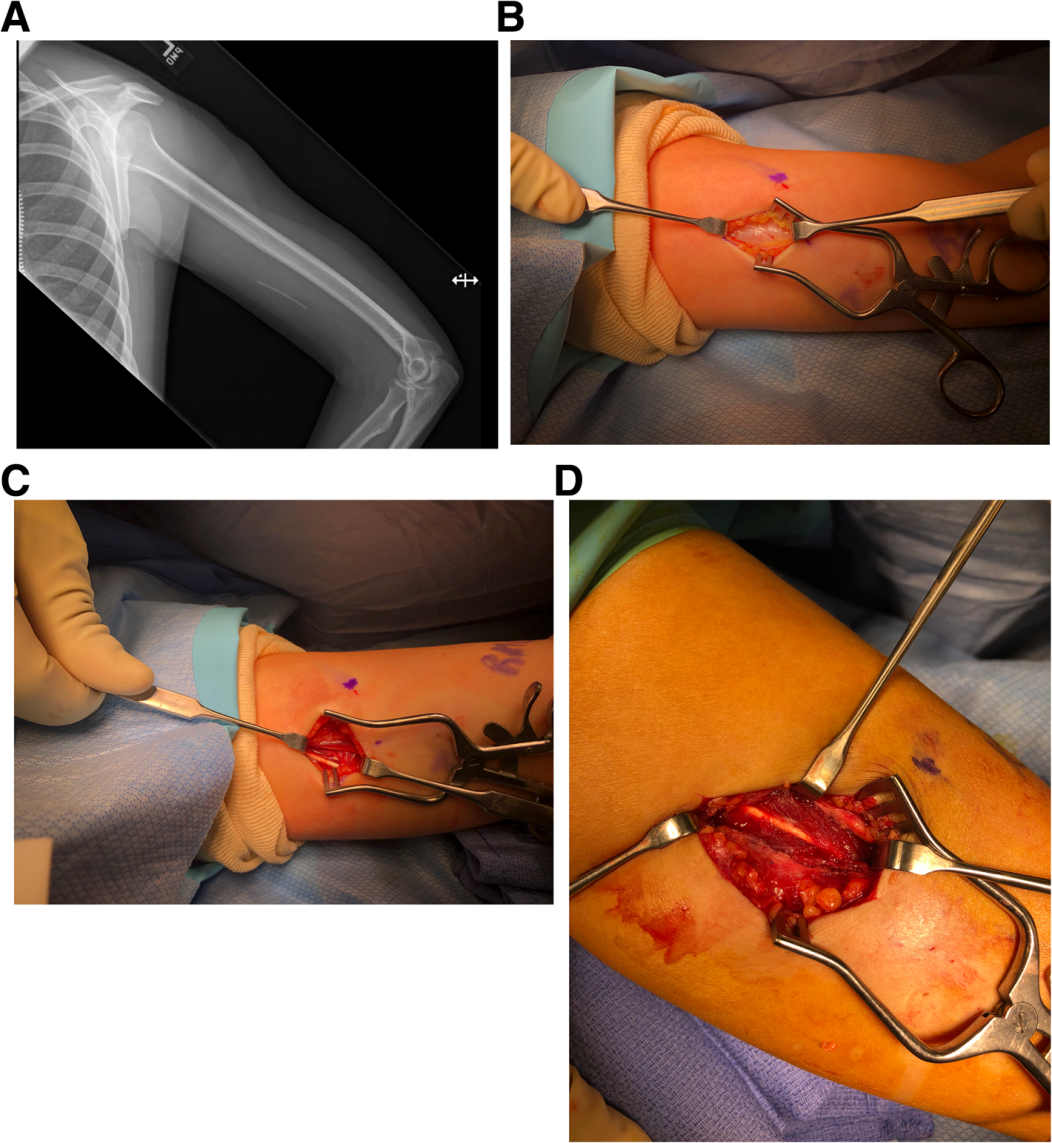
Radiographic identification of implanted contraceptive implant prior to incision in operating room (**a**). Incision and blunt dissection carried down to the fascial layer of the biceps brachii, which must be incised to retrieve the implant (**b**). Etonogestrel implant located deep to the biceps brachii fascia (**c**). Etonogestrel implant located within the muscle belly of the biceps brachii, surrounded by muscle fibers (**d**)

After IRB approval, we identified 6 cases of etonogestrel implants referred for surgical removal from 2018 to 2020 (Table 1). All patients had Nexplanon® implants placed within the preceding three years. The average age was 28 years (19–33) and BMI was 24.0 kg/m^2 (19.1–36.5). The most common reason for removal was irregular bleeding. We localized implants with XR (Fig. 1 a) and in-office high-frequency US to confirm deep location prior to referral to orthopaedic surgeon. Each implant was identified through physical examination by a qualified provider through palpation, XR and US guidance to determine the location within the soft tissue. For removal, a small incision and dissection clamp were utilized in attempt to retrieve the implants. Reasons for failure of in-office removal included close proximity to neurovascular structures, excessive pain with palpation concerning for neurologic irritation, and failure to adequately visualize the implants. Surgeons obtained informed consent after discussing the risks of medial upper arm exploration. Surgeons positioned patients supine with an arm table to allow adequate visualization of the upper extremity. Surgeons utilized conscious sedation and local anesthesia for all cases except one, where the patient preferred regional anesthesia. Intraoperative fluoroscopic guidance aided localization of the implant by its relative radiodensity within the soft tissue. Surgeons made a 2 to 7 centimeter incision longitudinally over the implant placement scar (Fig. 1b). Careful dissection was carried down through planes to expose the distal end of the implant. Implants were all located deep to the biceps brachii fascia or within the muscle belly itself (Fig. 1 c, d). The fascia and overlying soft tissue were then closed using suture. No intraoperative or postoperative complications occurred. The average procedure time from skin incision to closure was 14.3 min (Table 1).

**Table 1:** Characteristics of 6 patients undergoing removal of subfascial etonogestrel contraceptive implant by orthopaedic surgeons, including the length of time each patient had their implants, any attempts at in-office removal and surgical duration. One patient with prior implant transitioned to an alternative form of contraception before receiving her second implant and the other patient had her implant removed at the time of replacement of the most recent one. (BMI: Body mass index reported in kilogram/meters^2; MAC: Monitored Anesthesia Care)

AGE	BMI	Subfascial/Intramuscular	Duration (years)	First implant or prior?	Removal Attempts	Anesthesia	Procedure Duration (minutes)
31	19.1	Subfascial	2.96	First	Once	MAC/local	15
26	22.0	Subfascial	2.68	First	None	MAC/local	12
28	24.6	Subfascial	0.76	One prior (ipsilateral), removed in office	None	MAC/local	18
31	20.5	Subfascial	2.88	First	Once	MAC/local	11
33	36.5	Subfascial	1.02	First	None	Regional	10
19	21.5	Intramuscular	0.92	One prior (ipsilateral), removed in office	None	MAC/local	20
28	24.0						14.3 average

## Discussion/conclusion

This series presents 6 cases of etonogestrel implants located in a subfascial location requiring complex surgical removal by an upper extremity orthopaedic surgeon. Other series have identified subfascial location of contraceptive implants; very few described surgical extraction [3–5]. In our series, none of the implants were able to be retrieved in the office, despite subfascial exploration. Although not noted in this series, removal from subfascial planes has the potential for significant morbidity from larger incisions, anesthetic risk, and damage to nearby neurovascular structures [6, 7]. Rare cases describe migration of implants to the axilla and pulmonary vasculature, requiring more invasive surgical interventions [8–12].

The rarity of this condition precludes identification of specific risk factors for deep location. We noted no trends in prior implant use or removal attempts but 5 of 6 patients had normal BMI. Researchers theorize that low BMI may confer risk due to smaller distance between tissue planes in the upper arm [3]. Whether subfascial location alters contraceptive efficacy, side effects (e.g., bleeding profile) or duration of use warrants future study.

Incorrect placement of etonogestrel implants occurs in 12.6 per 1000 cases [13]. Alternate insertion sites in the skin overlying the scapula have been described [14]. The Food and Drug Administration requires a training session before providers can order implants; this training allows for as many placement and removal attempts as desired on a simulation arm. It is impossible to quantify whether providers do not complete training or are informally trained and how closely training mimics placement in a real arm. Adherence to recommendations to elevate the skin and perform placement at eye level may help avoid deep placement. All certified implant providers underwent mandatory retraining by June 1, 2019, to ensure placement in the ideal site 8–10 cm proximal to medial epicondyle and 3–5 cm posterior to the sulcus to avoid possible neurovascular injury [15]. All of the implants reported in this series were placed prior to this change in placement recommendation; future series should explore whether these new guidelines have minimized complications in placement.

Even with perfect technique, it is possible to insert the implant too deeply and the clinician or patient may not know until implant removal [9]. Informed consent at time of placement should include discussion of these rare complications. Initial attempt to remove the implant in the office setting is most optimized with plain radiography and ultrasound guidance, along with manual palpation to identify the location of the implant. Local anesthesia with lidocaine with epinephrine is typically adequate for this procedure, but providers may select a longer acting form of anesthesia if needed. If the implant is found to be within close proximity to neurovascular structures or deep within the soft tissue, there is considerable risk for injury to delicate structures due to poor visualization. These include superficial and deep sensory nerves, motor branches to muscles of the forearm and delicate vascular structures that can be difficult to visualize and control in a minimally invasive setting. Complex in-office removal and concern for neurovascular injury or inability to adequately localize the implant benefit from co-management with an upper extremity surgeon to safely remove intra-/subfascial implants.

## Data Availability

The data used to support the findings reported in this study are available by the corresponding author upon reasonable request.

## Abbreviations

XR: X-ray
US: Ultrasound
BMI: Body Mass Index

## Declarations

### Authors’ Information

PMC is a board-certified associate professor of obstetrics and gynecology at Columbia University Medical Center. She serves as the director of the faculty practice and specializes in contraceptive use in medically complex patients.

RKK is a dual fellowship-trained MD/PhD assistant professor of orthopedic surgery at Columbia University Medical Center. He specializes in upper extremity surgery and trauma.

## References

[1] Trussell J (2011). Contraceptive failure in the United States. Contraception.

[2] Merck & Co. I. Whitehouse Station NJ. Nexplanon. (etonogestrel implant) prescribing information https://www.merckcom/product/usa/pi_circulars/n/nexplanon/nexplanon_pipdf .[Accessed 17 June 2020].

[3] Matulich MC, Chen MJ, Schimmoeller NR, Hsia JK, Uhm S, Wilson MD (2019). Referral Center Experience With Nonpalpable Contraceptive Implant Removals. Obstet Gynecol.

[4] Petro G, Spence T, Patel M, Gertz AM, Morroni C. Difficult etonogestrel implant removals in South Africa: A review of 74 referred cases. Contraception. 2020. 10.1016/j.contraception.2020.04.013 PMC8221391 32339484

[5] Xu L, Korotkaya Y, Rible R. Deeply Inserted Contraceptive Implants: Where Are They Placed? Experience From a University Hospital Center. Obstetrics & Gynecology. 2020;135(p95S).

[6] Lefebvre R, Hom M, Leland H, Stevanovic M (2018). Peripheral nerve injury with Nexplanon removal: case report and review of the literature. Contracept Reprod Med.

[7] Jacques T, Henry S, Giraudet G, Demondion X, Cotten A (2020). Minimally-invasive fully ultrasound-guided removal of nonpalpable single-rod contraceptive implant: Case report and technical description. Contraception.

[8] Diego D, Tappy E, Carugno J (2017). Axillary migration of Nexplanon(R): Case report. Contraception.

[9] Kang S, Niak A, Gada N, Brinker A, Jones SC (2017). Etonogestrel implant migration to the vasculature, chest wall, and distant body sites: cases from a pharmacovigilance database. Contraception.

[10] Akhtar MM, Bhan A, Lim ZY, Akhtar MA, Sekhri N, Bharadwaj P (2018). Percutaneous extraction of an embolized progesterone contraceptive implant from the pulmonary artery. Open Access J Contracept.

[11] Gallon A, Fontarensky M, Chauffour C, Boyer L, Chabrot P (2017). Looking for a lost subdermal contraceptive implant? Think about the pulmonary artery. Contraception.

[12] Simon C, Maurier A, Gaboriau L, Vrignaud L, Dayani P, Vaillant T, et al. Incidence and characteristics of intravascular pulmonary migration of etonogestrel implants: A French nationwide study. Contraception. 2020. 10.1016/j.contraception.2020.05.006 32417204

[13] Reed S, Do Minh T, Lange JA, Koro C, Fox M, Heinemann K (2019). Real world data on Nexplanon(R) procedure-related events: final results from the Nexplanon Observational Risk Assessment study (NORA). Contraception.

[14] Pragout D, Darrouzain F, Marret H (2018). Alternative insertion site in the scapular region for etonogestrel contraceptive implant (Nexplanon((R))). Eur J Obstet Gynecol Reprod Biol.

[15] Iwanaga J, Fox MC, Rekers H, Schwartz L, Tubbs RS (2019). Neurovascular anatomy of the adult female medial arm in relationship to potential sites for insertion of the etonogestrel contraceptive implant. Contraception.

